# Epigenetic mechanisms of Nsd1-mediated histone methylation modifications in chondrocyte ferroptosis in knee osteoarthritis

**DOI:** 10.17305/bb.2024.10879

**Published:** 2024-08-31

**Authors:** Rao Wang, Da Shi, Xiaoni Pan, Anqi Ren, Kai Jiang

**Affiliations:** 1 Bone and Joint Rehabilitation Department of TCM Orthopedic Center, Honghui Hospital, Xi’an Jiaotong University, Xi’an, China; 2 Department of Clinical Pharmacy, Honghui Hospital, Xi’an Jiaotong University, Xi’an, China

**Keywords:** Knee osteoarthritis, ferroptosis, chondrocyte, Nsd1, Sox9, Acsl4

## Abstract

Knee osteoarthritis (KOA) is a degenerative joint disease characterized by pain, stiffness, and impaired mobility, with current therapies offering limited efficacy. This study investigates the epigenetic role of nuclear receptor-binding SET domain protein 1 (NSD1) in KOA pathogenesis. A KOA mouse model was established, and adenoviral vectors were employed to upregulate *Nsd1* and inhibit SRY-box transcription factor 9 (*Sox9*), followed by histopathological assessments. We examined changes in cell morphology, proliferation, viability, and ferroptosis-related markers. The expression of NSD1, SOX9, and acyl-CoA synthetase long-chain family member 4 (ACSL4) was analyzed, along with the enrichment of NSD1 and dimethylated lysine 36 of histone 3 (H3K36me2) on the *SOX9* promoter and SOX9 on the *ACSL4* promoter. Additionally, the binding relationship between SOX9 and the *ACSL4* promoter sequence was analyzed. Our results revealed that *NSD1* expression was reduced in KOA mouse tissues and interleukin-1β-stimulated chondrocytes. *NSD1* upregulation alleviated KOA, promoted chondrocyte proliferation and viability, and inhibited ferroptosis. Mechanistically, NSD1 enhanced H3K36me2 to upregulate *SOX9* expression, which in turn suppressed *ACSL4* expression and ferroptosis. *SOX9* inhibition partially reversed the protective effect of *NSD1* overexpression. In summary, *NSD1* upregulation mitigates chondrocyte ferroptosis and ameliorates KOA by modulating H3K36me2 to upregulate *SOX9* and downregulate *ACSL4* expression.

## Introduction

Osteoarthritis (OA) is a degenerative disease marked by pathological alterations in cartilage, bone, and synovial tissues, leading to joint dysfunction, pain, and reduced mobility [1]. Among the different forms of OA, knee OA (KOA) is particularly prevalent and is recognized as a multifactorial chronic disease with cartilage damage and aging being primary risk factors [2]. Traditionally, early OA treatment has focused on pain management and minimizing joint damage; however, the efficacy of both pharmacological and surgical interventions in achieving these objectives has been limited [3]. Recently, the role of ferroptosis, a unique form of iron-dependent cell death characterized by lipid peroxidation, has garnered significant attention in the context of OA pathogenesis [4]. Emerging evidence suggests that ferroptosis significantly contributes to chondrocyte death, reduced cell vitality, and degradation of the extracellular matrix (ECM), all of which are critical to the progression of OA [5, 6]. Notably, targeting chondrocyte ferroptosis has been proposed as a potential therapeutic strategy to mitigate cartilage injury in OA [7]. Thus, understanding the mechanisms of ferroptosis in KOA and identifying novel molecular targets for its treatment is of considerable significance.

Nuclear receptor-binding SET domain protein 1 (NSD1) is a histone methyltransferase encoded on human chromosome 5q35.3, responsible for catalyzing the mono-methylation and dimethylation of lysine 36 of histone 3 (H3K36) [8]. Previous studies have linked alterations in histone methylation, including at H3K36 and H3K27, to changes in gene expression and signaling pathways within chondrocytes affected by OA [9]. Moreover, it has been observed that NSD1-induced H3K36 methylation decreases in aged chondrocytes and cartilage affected by age-related OA [10]. Additionally, *Nsd1* has been shown to play a crucial role in cartilage formation and chondrocyte differentiation [11]. However, the specific involvement of NSD1 in regulating chondrocyte ferroptosis via histone modifications remains unexplored.

The SRY-box transcription factor 9 (SOX9) gene is crucial for ECM production in chondrocytes, playing a vital role in maintaining chondrocyte homeostasis and promoting early differentiation [12]. SOX9 activates the expression of key cartilage components, such as type II collagen alpha1 chain (Col2a1) and aggrecan (Acan), both of which are essential for articular cartilage (AC) development [13]. Interestingly, a reduction in *SOX9* protein expression has been associated with interleukin (IL)-1β-induced ferroptosis in chondrocytes [14]. Our study delves deeper into the interaction between SOX9 and Nsd1, exploring how this relationship influences ferroptosis pathways.

Acyl-CoA synthetase long-chain family member 4 (ACSL4) is a protein-coding gene involved in lipid metabolism, signal transduction, and the regulation of ferroptosis [15]. ACSL4 is known to promote lipid peroxidation, particularly in the absence of glutathione peroxidase 4 (GPX4), thereby driving ferroptosis [16]. Inhibition of *Acsl4* has been shown to reduce reactive oxygen species (ROS) and malondialdehyde (MDA), a key byproduct of lipid peroxidation, thereby mitigating ferroptosis in chondrocytes [17]. Through database analysis, we identified a potential binding interaction between SOX9 and *ACSL4*, leading us to hypothesize that the NSD1/SOX9/ACSL4 axis may represent a novel therapeutic target for KOA.

In this study, we established both a mouse model of KOA and a human chondrocyte model to investigate the mechanisms by which Nsd1 regulates chondrocyte ferroptosis via the SOX9/ACSL4 axis and histone modifications. Our findings aim to provide a theoretical basis for the development of new therapeutic strategies for KOA.

## Materials and methods

### Laboratory animals

Healthy male C57BL/6 mice (*n* ═ 72, aged 8–10 weeks, weighing 20–23 g) were obtained from Beijing Vital River Laboratory Animal Technology (Beijing, China). The mice were acclimatized for one week in a specific pathogen-free environment with standard lighting and were provided unrestricted access to food and water.

### KOA model establishment and treatment

The KOA mouse model was established as previously described [18]. Mice were intraperitoneally anesthetized with 2% sodium pentobarbital (2 mL/kg). The surgical area was thoroughly disinfected before making a medial parapatellar incision on the right knee to fully flex the knee joint. The anterior horn of the medial meniscus was exposed and incised, followed by resection to separate it from the medial side. Under direct visualization, the anterior cruciate ligament (ACL) was amputated, and complete transection was confirmed by the anterior drawer test. Throughout the procedure, the AC surfaces were protected. The joint cavity was irrigated with saline, and the joint capsule and skin were sutured in layers. Mice in the sham group underwent only a skin incision without further intervention.

Adenoviruses, including Ad-Nsd1, Ad-NC (empty plasmid without target gene), Ad-sh-Sox9, and the corresponding Ad-sh-NC, were constructed and packaged by GenePharma (Shanghai, China). Adenoviruses (5 × 10^9^ pfu) were injected into the knee joints of mice 24 h before inducing OA. All mice were euthanized eight weeks after OA modeling by intraperitoneal injection of 200 mg/kg pentobarbital. Post-euthanasia, the mice were positioned supine, with forelimbs secured, and the skin and soft tissues of the hindlimbs were removed to expose the knee joints. The tibial plateau was dissected, and the regular translucent sphere-like appearance of the joint surface was preserved. The AC tissues were fixed in 4% paraformaldehyde, decalcified in 10% ethylene diamine tetraacetic acid (EDTA), dehydrated through an ethanol gradient, and permeabilized with xylene. Paraffin-embedded sections were cut into 5 µm thick slices using a microtome (CUT4060, Leica, Germany). The sections were deparaffinized and stained using hematoxylin and eosin (H&E) staining, safranin O-fast green staining, and immunohistochemistry. Additional AC tissues were collected and stored in liquid nitrogen for subsequent analysis, including enzyme-linked immunosorbent assay (ELISA), reverse transcription-quantitative polymerase chain reaction (RT-qPCR), and Western blot analysis.

### Tissue staining

H&E staining was used to assess the histopathological status of the AC. Briefly, AC tissue sections were stained with hematoxylin (A600701-0010; Sangon Biotech, Shanghai, China), followed by differentiation with 1% hydrochloric acid alcohol and treatment with 1% ammonia water. The sections were then counterstained with 1% eosin solution (A600440-0025; Sangon Biotech). Subsequently, the AC tissue sections were dehydrated and cleared using a graded ethanol series (75%, 90%, and 95% ethanol) followed by xylene (two changes). Finally, the sections were dried and mounted. The morphology and structure of the AC were examined under a light microscope.

Safranin O staining was performed to evaluate the damage to AC tissue. Tissue sections were stained with Weigert’s iron hematoxylin, followed by staining with 0.2% fast green solution (C500016-0500; Sangon Biotech), 1% acetic acid solution, and 0.1% Safranin O solution (A600815-0025; Sangon Biotech). The sections were then dehydrated, cleared, and mounted with neutral balsam. Cartilage degeneration was assessed by three independent investigators using the Osteoarthritis Research Society International (OARSI) grading method [19], with ranges from Grade 0 (normal) to Grade 6: intact cartilage and surface, Grade 0; intact surface, Grade 1; surface discontinuity, Grade 2; vertical fracture, Grade 3; erosion, Grade 4; denudation, Grade 5; and deformation, Grade 6.

### Cell culture and treatment

The human immortalized chondrocyte cell line C-28/I2 (cat. SCC043) was purchased from Sigma-Aldrich (St. Louis, MI, USA) and cultured in Dulbecco’s modified Eagle medium (DMEM)/Ham’s F12 medium (R&D systems, Minneapolis, MN, USA) supplemented with 10% fetal bovine serum (R&D systems). Cells were maintained in a humidified incubator at 37 ^∘^C with 5% CO_2_. When C-28/I2 cells reached approximately 70% confluence, they were transiently transfected with exogenous nucleic acids using Lipofectamine 3000 (Invitrogen, Carlsbad, CA, USA) according to the manufacturer’s instructions. To silence SOX9, small interfering RNAs (siRNAs) targeting SOX9 (si-SOX9-1, si-SOX9-2, and si-SOX9-3) were transfected into the cells. For overexpression of NSD1, the pcDNA-NSD1 (oe-NSD1) vectors carrying NSD1 complementary DNA (cDNA) were transfected. Negative control siRNAs (si-NC) and empty vector controls (oe-NC) were also used, all of which were synthesized by GenePharma. After 36 h of transfection, the cells were harvested for total protein/RNA isolation or subjected to IL-1β treatment.

To induce an OA-like phenotype, recombinant human IL-1β (Sigma-Aldrich) at a concentration of 10 ng/mL was added to C-28/I2 cells at approximately 80% confluence for 24 h, mimicking chondrocyte damage as seen in OA. Cells treated with an equal volume of phosphate-buffered saline (PBS; pH 7.4) served as controls for IL-1β treatment.

### Measurement of glutathione (GSH), MDA, and Fe^2+^ levels

Cartilage tissue and chondrocytes were lysed using cell lysis buffer (Solarbio, Beijing, China). The concentrations of MDA and GSH were measured using commercial assay kits from Beyotime (Shanghai, China). Fe^2+^ levels were detected using an iron assay kit (Dojindo, Japan) according to the manufacturer’s instructions.

### ROS detection

The levels of ROS in cells and tissue homogenates were measured using 2′,7′-dichlorodihydrofluorescein diacetate (DCFH-DA) dye (S0033S, Beyotime) following the manufacturer’s instructions. Cells or tissue sections were incubated with 10 µM DCFH-DA at 37 ^∘^C. After washing with PBS, fluorescence was detected using a fluorescence microscope (EVOS FL Auto, Thermo Fisher Scientific, Waltham, MA, USA).

### Cell Counting Kit-8 (CCK-8) assay

Chondrocytes were cultured in 96-well plates. After 24 h, the culture medium was removed, and 100 µL of 10% CCK-8 solution was added to each well. The cells were incubated at 37 ^∘^C in the dark for 1 h. The optical density was measured at 450 nm using a microplate reader (Thermo Fisher Scientific).

### Quantitative real-time polymerase chain reaction (qRT-PCR)

Total RNA was extracted from cartilage tissues and chondrocytes using an RNA purification kit (Takara, China). Reverse transcription was performed with a reverse transcription kit (Takara), and qPCR was conducted using qPCR Mix (Takara) according to the manufacturer’s instructions. Gene expression levels were calculated using the 2^−ΔΔCt^ method [20] and normalized to glyceraldehyde-phosphate dehydrogenase (*GAPDH*). Primer sequences are shown in Table 1.

**Table 1:** PCR primer sequences

**Gene**	**Sequences (5′–3′)**
*NSD1* (human)	F: GAGCTACCTGTCCTTAGGAGAA
	R: GACTCAGGATCATTTGTGCAGT
*Nsd1* (mouses)	F: ATTTGGGCAAAATTCAAGAGACG
	R: GCCTCCTATTGGCAACTTTCATT
*SOX9* (human)	F: AGCGAACGCACATCAAGAC
	R: CTGTAGGCGATCTGTTGGGG
*Sox9* (mouses)	F: GAGCCGGATCTGAAGAGGGA
	R: GCTTGACGTGTGGCTTGTTC
*ACSL4* (human)	F: CATCCCTGGAGCAGATACTCT
	R: TCACTTAGGATTTCCCTGGTCC
*Acsl4* (mouses)	F: CTCACCATTATATTGCTGCCTGT
	R: TCTCTTTGCCATAGCGTTTTTCT
*GAPDH* (human)	F: GGAGCGAGATCCCTCCAAAAT
	R: GGCTGTTGTCATACTTCTCATGG
*Gapdh* (mouses)	F: AGGTCGGTGTGAACGGATTTG
	R: TGTAGACCATGTAGTTGAGGTCA
*SOX9* promoter (human)	F: AGAGTTTCCCAATGCTGTGC
	R: AAAAGTCTTGGTCGCTTGCG
*Sox9* promoter (mouses)	F: TTGCAAACTTCGCCCTCAAC
	R: TTCTGCTTCTTGCCTCCTGAC
*ACSL4* promoter (human)	F: GTCTTAGCAATATTTATAGG
	R: TTCTGATCTGGGTAGGGATAA
*Acsl4* promoter (mouses)	F: CCAGGTATTCCCATTGGAACAA
	R: TTCTCTTACAGAATGACTGTGT

Nsd1: Nuclear receptor-binding SET domain protein 1; SOX9: SRY-box transcription factor 9; ACSL4: Acyl-CoA synthetase long-chain family member 4; GAPDH: Glyceraldehyde-phosphate dehydrogenase.

### Western blot assay

Total proteins from cartilage tissues and chondrocytes were extracted using radioimmunoprecipitation assay (RIPA) lysis buffer (Beyotime). Proteins were separated on sodium dodecyl sulfate-polyacrylamide gel electrophoresis (SDS-PAGE) gels (Willget, Shanghai, China) and transferred to nitrocellulose membranes (Pall, Washington, NY, USA). The membranes were incubated overnight with primary antibodies against NSD1 (1:1000, PA5-50857, Invitrogen), GPX4 (1:5000, ab125066, Abcam, Cambridge, MA, USA), ACSL4 (1:10000, ab155282, Abcam), SOX9 (1:1000, 702016, Invitrogen), solute carrier family 7 member 11 (SLC7A11) (1:1000, PA1-16893, Invitrogen), and MMP13 or β-actin (1:5000, ab8227, Abcam). After incubation with secondary antibodies (1:5000, ab6721, Abcam), the blots were visualized using an enhanced chemiluminescence kit (Beyotime).

### Chromatin immunoprecipitation (ChIP)

ChIP assays were performed using the Simple ChIP^®^ Enzymatic ChIP Kit (9003s, Cell Signaling Technologies). Samples were initially treated with 1% formaldehyde at room temperature for 10 min to crosslink proteins to DNA, followed by the addition of glycine to quench the reaction for 5 min. DNA fragmentation was conducted as per the kit’s instructions. The immunoprecipitated chromatin complexes were then probed with antibodies against NSD1 (1:100, PA5-50857, Invitrogen), dimethylated lysine 36 of histone 3 (H3K36me2) (1:500, ab176921, Abcam), SOX9 (1:1000, 702016, Invitrogen), or a negative control rabbit immunoglobulin G (IgG) (1:100, NBP2-24891, NOVUS, Littleton, Co, USA). Following de-crosslinking with NaCl and proteinase K, DNA was extracted. The enrichment levels of NSD1 and H3K36me2 on the *SOX9* promoter, as well as SOX9 on the *ACSL4* promoter, were quantified using qPCR. Primer sequences used for qPCR are listed in Table 1.

### Dual-luciferase reporter assay

The binding sites between SOX9 and the *ACSL4* promoter were identified using the JASPAR database. Synthesized *ACSL4* promoter fragments containing the binding site for SOX9 (ACSL4-WT) and mutated binding sites (ACSL4-MUT) were cloned into reporter plasmids (Beijing Huayueyang Biotechnology, Beijing, China) to generate ACSL4-WT and ACSL4-MUT constructs, respectively. These luciferase reporter plasmids were co-transfected with either oe-NC or oe-SOX9 (GenePharma) into C-28/I2 cells. After 48 h of transfection, cells were harvested and lysed. Luciferase activity was measured using a luciferase assay kit (R&D system) according to the manufacturer’s instructions. All experiments were conducted in triplicate.

### Ethical statement

All animal experiments were approved by the Institutional Animal Care and Use Committee of Xi’an Honghui Hospital (Approval No.: 202303054) and were conducted in accordance with the Guide for the Care and Use of Laboratory Animals [21].

### Statistical analysis

Data were analyzed and visualized using SPSS Statistics 21.0 (IBM, Armonk, NY, USA) and GraphPad Prism 8.0 (GraphPad Software Inc., San Diego, CA, USA). Normality and homogeneity of variance were first assessed to ensure that the data met the assumptions for parametric tests. Comparisons between two groups were conducted using the *t*-test. For multiple group comparisons, one-way or two-way analysis of variance (ANOVA) was applied, followed by Tukey’s multiple comparisons test for post hoc analysis. *P* values were obtained from two-sided tests; statistical significance was set at *P* < 0.05 and highly significant differences indicated by *P* < 0.01.

## Results

### Overexpression of *Nsd1* alleviates symptoms of KOA and cell ferroptosis in mice

**Figure 1.**
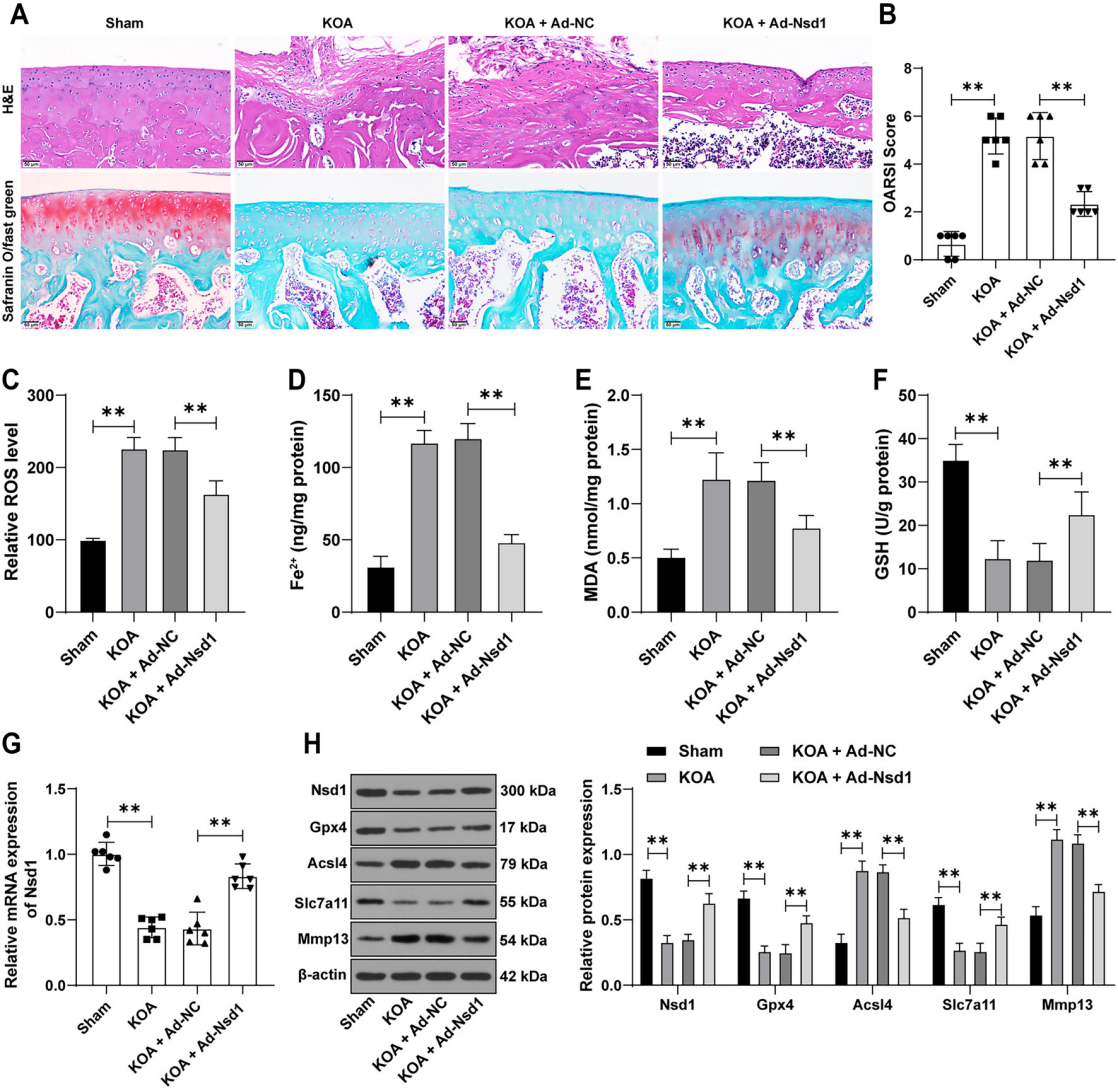
**Overexpression of *Nsd1* alleviates symptoms of KOA and cell ferroptosis in mice.** The expression of *Nsd1* was upregulated in the mouse knee joints using an adenovirus-packaged *Nsd1* overexpression vector (Ad-Nsd1), with an adenovirus carrying an empty vector (Ad-NC) used as a control. One day after surgery, a KOA mouse model was established, and after eight weeks, the mice were euthanized, and knee joint cartilage tissues were collected. (A) Tissue pathological changes were observed through H&E staining and safranin O/fast green staining; (B) The OARSI score was used to assess the degree of injury; (C) ROS levels were measured using DCFH-DA fluorescence labeling; (D–F) The levels of Fe^2+^, MDA, and GSH were detected using specific assay kits; (G) The expression of *Nsd1* in cartilage tissue was evaluated using qRT-PCR; (H) The expression of NSD1, GPX4, ACSL4, SLC7A11, and MMP13 in cartilage tissue was examined using a Western blot assay. Data in panels (B)–(G) were analyzed using one-way ANOVA, followed by Tukey’s multiple comparisons test. Data in panel (H) were analyzed using two-way ANOVA, followed by Tukey’s multiple comparisons test. **P < 0.01. Nsd1: Nuclear receptor-binding SET domain protein 1; KOA: Knee osteoarthritis; NC: Negative control; H&E: Hematoxylin and eosin; OARSI: Osteoarthritis Research Society International; ROS: Reactive oxygen species; DCFH-DA: 2′,7′-dichlorodihydrofluorescein diacetate; MDA: Malondialdehyde; GSH: Glutathione; qRT-PCR: Quantitative real-time polymerase chain reaction; Gpx4: Glutathione peroxidase 4; Acsl4: Acyl-CoA synthetase long-chain family member 4; Slc7a11: Solute carrier family 7 member 11; Mmp13: Matrix metalloprotease 13; ANOVA: Analysis of variance.

In our study, a mouse model of KOA was successfully established, revealing severe cartilage damage, fibrosis, thickening, and irregularities in the AC. The OARSI score was significantly elevated in KOA mice compared to control mice (*P* < 0.01, Figure 1A and 1B), along with an increase in Mmp13 expression (*P* < 0.01, Figure 1H). Analysis of ferroptosis in the cartilage tissue showed a significant rise in levels of ROS, Fe^2+^, MDA, and ACSL4 in KOA mice (*P* < 0.01, Figure 1C–E and 1H), while levels of GSH, GPX4, and SLC7A11 were markedly reduced (*P* < 0.01, Figure 1F and 1H). Notably, *Nsd1* expression was significantly decreased in KOA mice (*P* < 0.01, Figure 1G and 1H). To investigate the role of *Nsd1* in KOA, we overexpressed *Nsd1* in the KOA mouse model (*P* < 0.01, Figure 1G and 1H). *Nsd1* overexpression significantly ameliorated the pathological changes observed in KOA tissues (*P* < 0.01, Figure 1A), reduced the OARSI score (*P* < 0.01, Figure 1B), decreased MMP13 expression (*P* < 0.01, Figure 1H), and mitigated ferroptosis in the tissues (*P* < 0.01, Figure 1C–1F and 1H). These findings indicate that *Nsd1* overexpression plays a protective role in alleviating KOA and reducing ferroptosis in mice.

### Overexpression of *NSD1* alleviates IL-1**β**-induced chondrocyte ferroptosis

**Figure 2.**
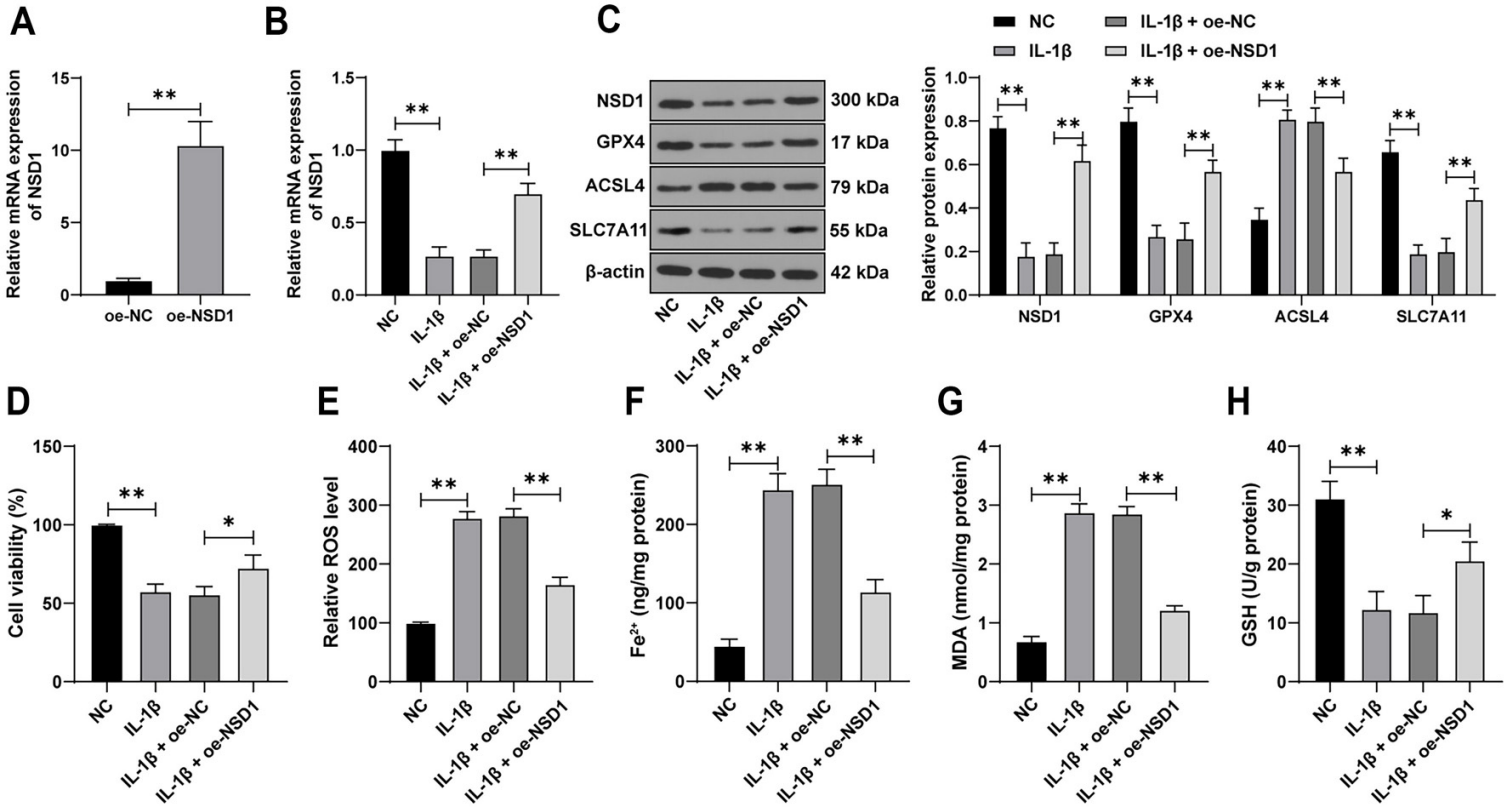
**Overexpression of *NSD1* alleviates IL-1β-induced chondrocyte ferroptosis.** C-28/I2 cells were cultured *in vitro*, and the overexpression vector for *NSD1* (oe-NSD1) was transfected into the cells, with an empty vector (oe-NC) used as a control. (A) Transfection efficiency was determined by qRT-PCR. IL-1β treatment was added to simulate the KOA condition; (B) The expression of *NSD1* was measured by qRT-PCR; (C) The expression of NSD1, GPX4, ACSL4, and SLC7A11 in C-28/I2 cells was examined by Western blot assay; (D) Cell viability was assessed using the CCK-8 assay; (E) ROS levels were measured using DCFH-DA fluorescence labeling; (F–H) The levels of Fe^2+^, MDA, and GSH were detected using specific assay kits. The cell experiments were repeated three times. Data in panel (A) were analyzed using a *t*-test. Data in panels (B) and (D–H) were analyzed using one-way ANOVA, followed by Tukey’s multiple comparisons test. Data in panel (C) were analyzed using two-way ANOVA, followed by Tukey’s multiple comparisons test. *P < 0.05, **P < 0.01. NSD1: Nuclear receptor-binding SET domain protein 1; IL: Interleukin; KOA: Knee osteoarthritis; NC: Negative control; ROS: Reactive oxygen species; CCK-8: Cell Counting Kit-8 DCFH-DA: 2’,7’-dichlorodihydrofluorescein diacetate; MDA: Malondialdehyde; GSH: Glutathione; qRT-PCR: Quantitative real-time polymerase chain reaction; GPX4: Glutathione peroxidase 4; ACSL4: Acyl-CoA synthetase long-chain family member 4; SLC7A11: Solute carrier family 7 member 11; ANOVA: Analysis of variance.

To further explore the effects of *NSD1* in an *in vitro* KOA model, we treated the human chondrocyte cell line C-28/I2 with IL-1β to simulate the KOA environment. IL-1β treatment led to a significant reduction in *NSD1* expression (*P* < 0.01, Figure 2B and 2C). Transfection with *NSD1* overexpression vectors successfully upregulated *NSD1* expression in C-28/I2 cells (*P* < 0.01, Figure 2A–2C). This upregulation of *NSD1* restored cell viability in IL-1β-induced C-28/I2 cells (*P* < 0.05, Figure 2D), reduced levels of ROS, Fe^2+^, MDA, and ACSL4 (*P* < 0.01, Figure 2C and 2E–2G), and increased levels of GSH, GPX4, and SLC7A11 (*P* < 0.05, Figure 2C and 2H). These findings indicate that *NSD1* overexpression effectively inhibits chondrocyte ferroptosis *in vitro*.

### NSD1 promotes the expression of *SOX9* through H3K36me2 modification

**Figure 3.**
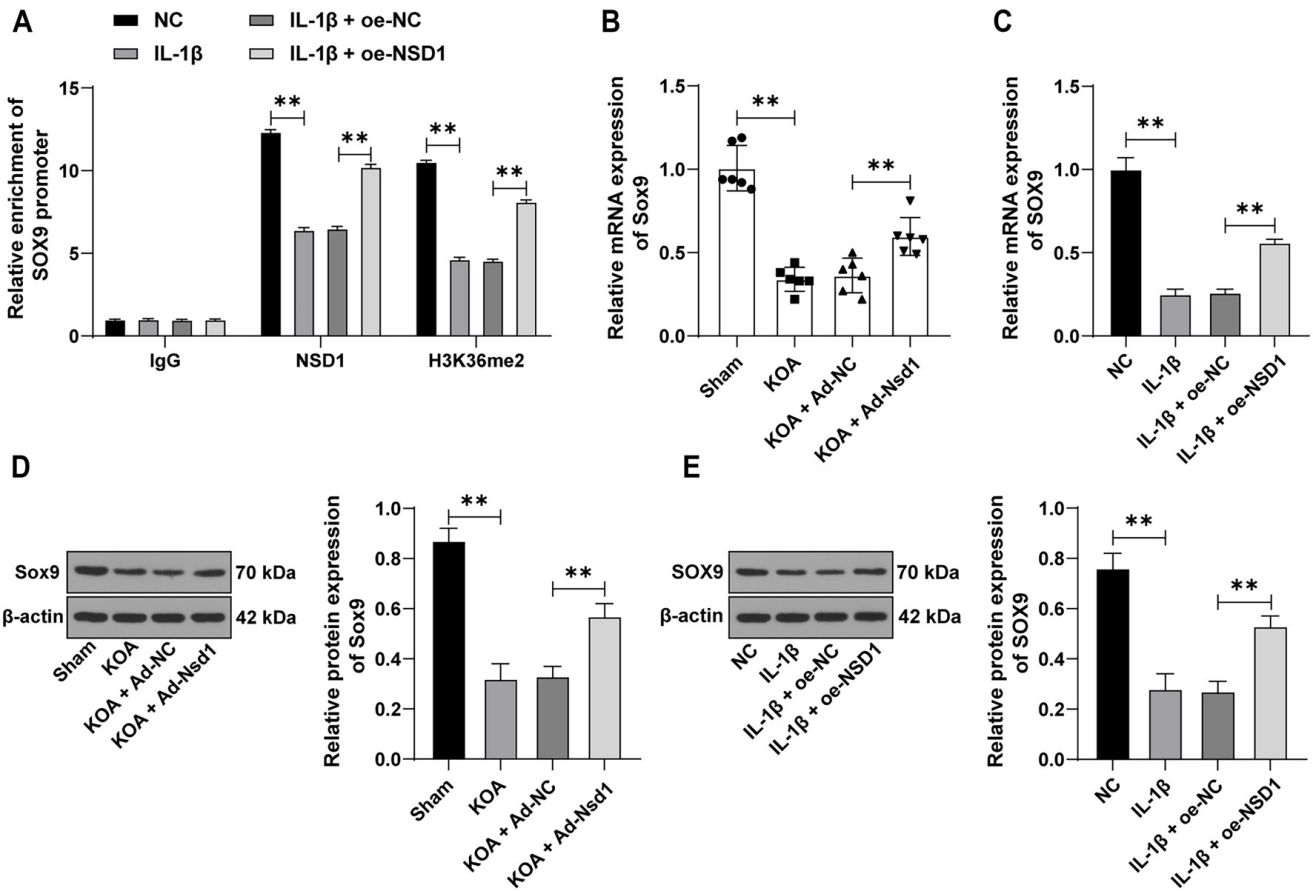
***NSD1* promotes the expression of *SOX9* through H3K36me2 modification.** (A) ChIP experiments were performed to analyze the enrichment of NSD1 and H3K36me2 on the *SOX9* promoter in chondrocytes; (B and C) qRT-PCR was used to detect the expression of *SOX9* in tissues (*n* ═ 6) and cells (*n* ═ 3); (D and E) Western blot assay was conducted to examine the expression of SOX9 in tissues (*n* ═ 6) and cells (*n* ═ 3). Data in panel (A) were analyzed using two-way ANOVA. Data in panels (B–E) were analyzed using one-way ANOVA, followed by Tukey’s multiple comparisons test. **P < 0.01. NSD1: Nuclear receptor-binding SET domain protein 1; SOX9: SRY-box transcription factor 9; H3K36me2: Dimethylate lysine 36 of histone 3; ChIP: Chromatin immunoprecipitation; qRT-PCR: Quantitative real-time polymerase chain reaction; ANOVA: Analysis of variance.

Previous research has demonstrated that NSD1 regulates *SOX9* expression via H3K36me2 modification, contributing to chondrocyte differentiation [11]. Since *SOX9* is known to be downregulated in OA [14], we hypothesized that NSD1 could upregulate *SOX9* expression by enhancing H3K36me2 modification. ChIP assays confirmed the enrichment of NSD1 and H3K36me2 on the *SOX9* promoter, which significantly increased following *NSD1* overexpression (*P* < 0.01, Figure 3A). Additionally, both the transcription and protein levels of *SOX9* were upregulated with the enhanced enrichment of NSD1 and H3K36me2 (*P* < 0.01, Figure 3B–3E). These findings suggest that NSD1 promotes *SOX9* expression by enhancing H3K36me2 modification.

### Inhibition of *SOX9* partially reverses the inhibitory effect of *NSD1* overexpression on ferroptosis in chondrocytes

**Figure 4.**
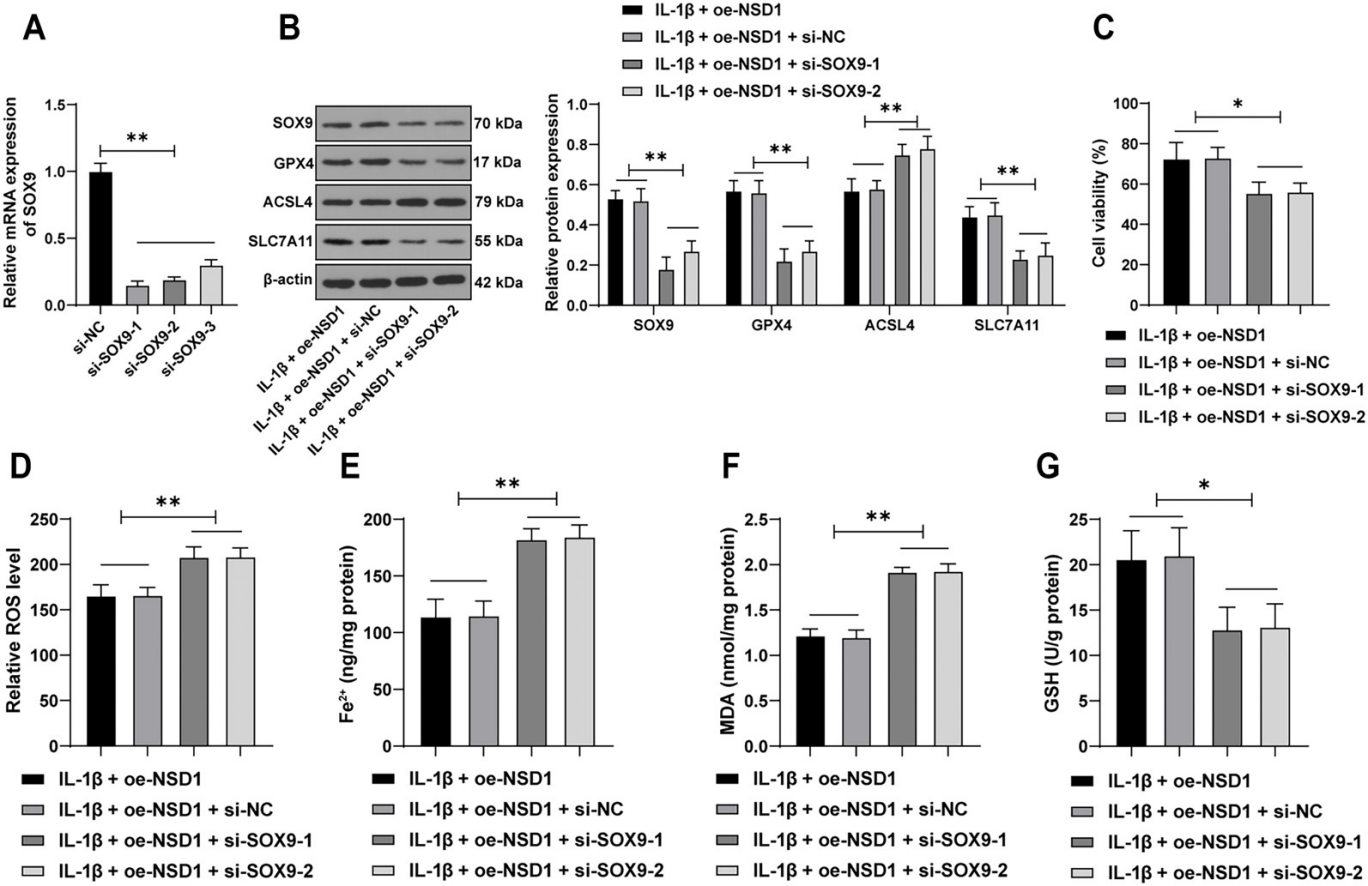
**Inhibition of *SOX9* partially reverses the inhibitory effect of *NSD1* overexpression on ferroptosis in chondrocytes.** C-28/I2 cells were transfected with three different si-SOX9 sequences (si-SOX9-1, si-SOX9-2, and si-SOX9-3) to suppress the expression of *SOX9*, with si-NC used as a control. (A) Transfection efficiency was determined by qRT-PCR. Two si-SOX9 sequences with good transfection efficiency were selected for co-transfection with oe-NSD1; (B) The expression of NSD1, GPX4, ACSL4, and SLC7A11 in C-28/I2 cells was examined by Western blot assay; (C) Cell viability was assessed using the CCK-8 assay; (D) ROS levels were measured using DCFH-DA fluorescence labeling; (E–G) The levels of Fe^2+^, MDA, and GSH were detected using specific assay kits. The cell experiments were repeated three times. Data in panels (A) and (C–G) were analyzed using one-way ANOVA, followed by Tukey’s multiple comparisons test. Data in panel (B) were analyzed using two-way ANOVA, followed by Tukey’s multiple comparisons test. *P < 0.05, **P < 0.01. NSD1: Nuclear receptor-binding SET domain protein 1; NC: Negative control; ROS: Reactive oxygen species; DCFH-DA: 2’,7’-dichlorodihydrofluorescein diacetate; MDA: Malondialdehyde; GSH: Glutathione; qRT-PCR: Quantitative real-time polymerase chain reaction; GPX4: Glutathione peroxidase 4; ACSL4: Acyl-CoA synthetase long-chain family member 4; SLC7A11: Solute carrier family 7 member 11; CCK-8: Cell Counting Kit-8; ANOVA: Analysis of variance; SOX9: SRY-box transcription factor 9; oe-NSD1: Overexpression vector for NSD1.

To further investigate the role of *SOX9* in the NSD1-mediated inhibition of ferroptosis, we transfected three siRNA constructs targeting SOX9 (si-SOX9) into C-28/I2 chondrocytes, successfully suppressing *SOX9* expression (*P* < 0.01, Figure 4A and 4B). The two most effective si-SOX9 constructs were selected for subsequent experiments involving co-transfection with the NSD1 overexpression vector. Compared to cells overexpressing *NSD1* alone, *SOX9* suppression led to a significant decrease in cell viability (*P* < 0.05, Figure 4C). Additionally, levels of ROS, Fe^2+^, MDA, and ACSL4 were significantly increased (*P* < 0.01, Figure 4B and 4D–4F), while levels of GSH, GPX4, and SLC7A11 were reduced (*P* < 0.05, Figure 4B and 4G). These findings suggest that inhibition of *SOX9* partially reverses the protective effect of *NSD1* overexpression on ferroptosis in chondrocytes.

**Figure 5.**
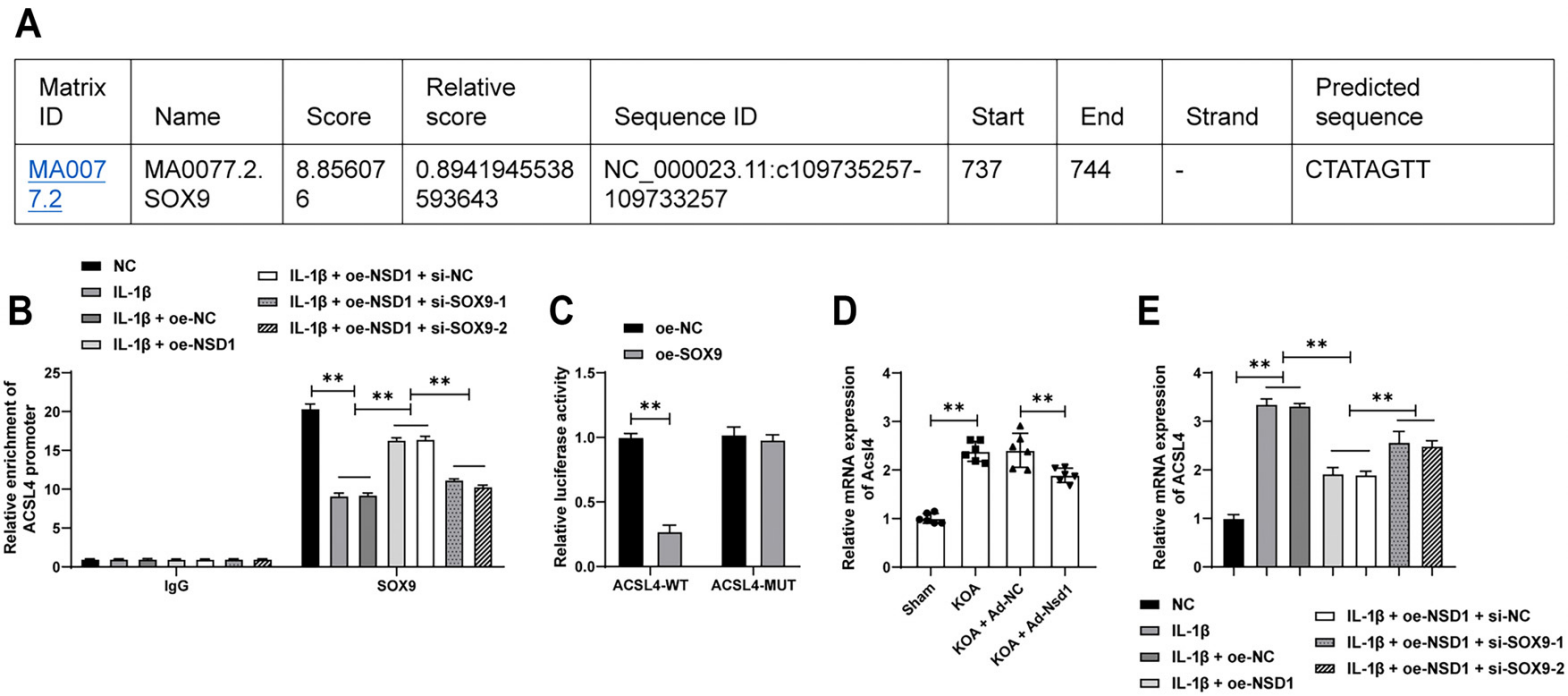
**SOX9 suppresses the transcription of *ACSL4*.** (A) The binding sites of SOX9 on the *ACSL4* promoter sequence were analyzed using the JASPAR database; (B) ChIP experiments were performed to analyze the enrichment of SOX9 on the ACSL4 promoter; (C) Dual luciferase reporter assay was conducted to investigate the binding relationship between SOX9 and the *ACSL4* promoter sequence; (D and E) qRT-PCR was used to detect the expression of ACSL4 in tissues (*n* ═ 6) and cells (*n* ═ 3). The cell experiments were repeated three times. Data in panels (B and C) were analyzed using two-way ANOVA. Data in panels (D and E) were analyzed using one-way ANOVA, followed by Tukey’s multiple comparisons test. **P < 0.01. SOX9: SRY-box transcription factor 9; ACSL4: Acyl-CoA synthetase long-chain family member 4; ChIP: Chromatin immunoprecipitation; qRT-PCR: Quantitative real-time polymerase chain reaction; ANOVA: Analysis of variance.

### *SOX9* suppresses *ACSL4* transcription

Further bioinformatics analysis using the JASPAR database identified putative SOX9 binding sites within the *ACSL4* promoter region (Figure 5A). This interaction was confirmed through ChIP analysis and dual-luciferase reporter assays, which demonstrated significant binding of SOX9 to the *ACSL4* promoter (*P* < 0.01, Figure 5B and 5C). Moreover, increased SOX9 enrichment at the *ACSL4* promoter led to a suppression of *ACSL4* transcription levels (*P* < 0.01, Figure 5D and 5E). These findings indicate that SOX9 acts as a transcriptional repressor of *ACSL4* by directly binding to its promoter.

### Inhibition of *Sox9* partially reverses the protective effect of *Nsd1* overexpression on KOA symptoms in mice

**Figure 6.**
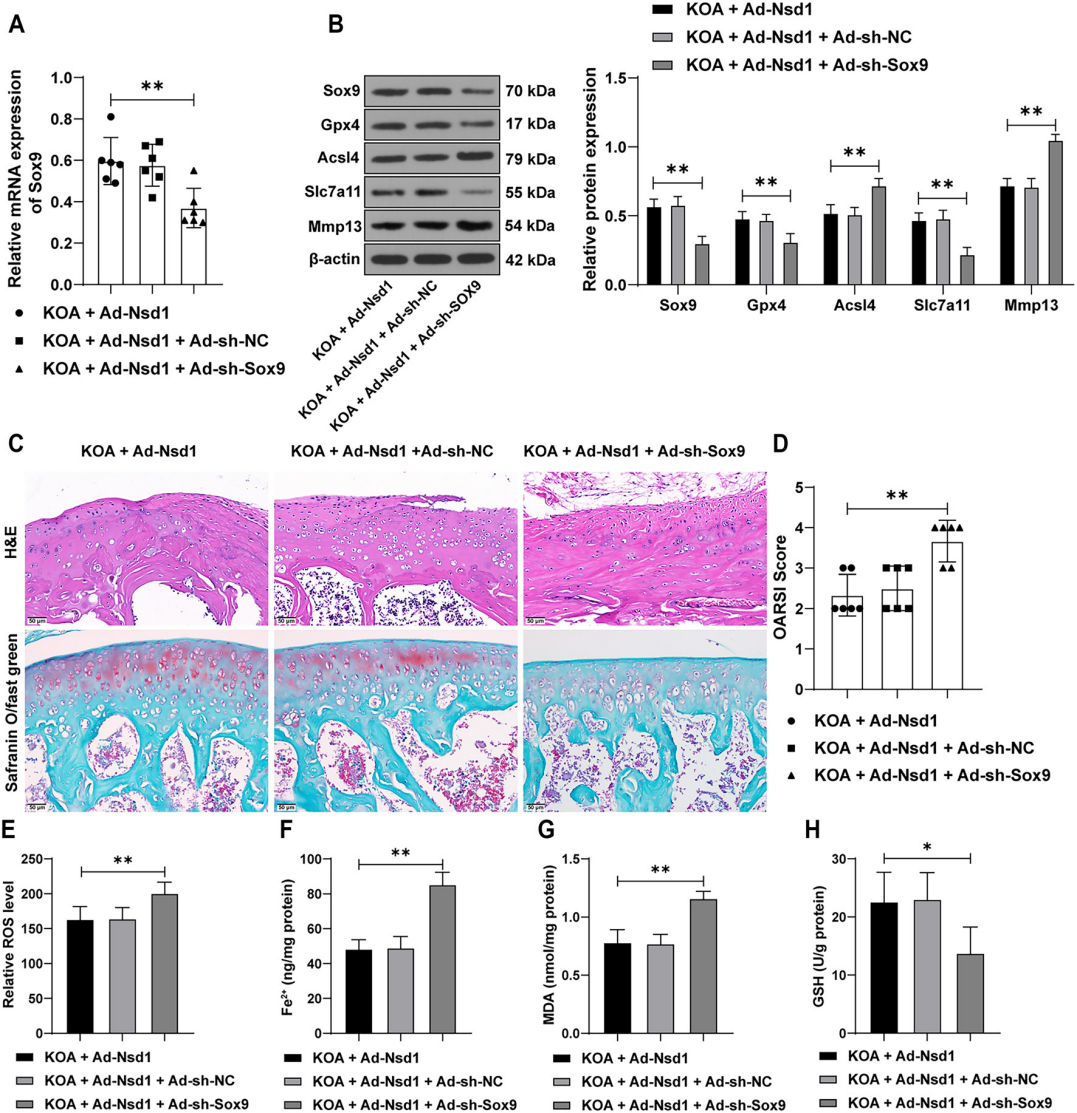
**Inhibition of *Sox9* partially reverses the relief effect of *Nsd1* overexpression on KOA symptoms in mice.** Ad-sh-Sox9, packaged in adenovirus was used to inhibit the expression of *Sox9* in the knee joints of mice, with Ad-sh-NC used as a control. One day after surgery, a mouse model of KOA was established. After eight weeks, the mice were euthanized, and knee joint cartilage tissues were collected. (A) qRT-PCR was performed to detect the expression of *Sox9* in the cartilage tissues; (B) The expression of SOX9, GPX4, ACSL4, SLC7A11, and MMP13 in the cartilage tissues was analyzed by Western blot assay; (C) Tissue pathological changes were observed through H&E staining and safranin O/fast green staining; (D) The OARSI score was used to assess the damage; (E) ROS levels were measured using DCFH-DA fluorescence labeling; (F–H) The levels of Fe^2+^, MDA, and GSH were detected using specific assay kits. *n* ═ 6. Data in panels (A) and (D–H) were analyzed using one-way ANOVA, followed by Tukey’s multiple comparisons test. Data in panel (B) were analyzed using two-way ANOVA, followed by Tukey’s multiple comparisons test. *P < 0.05, **P < 0.01. Nsd1: Nuclear receptor-binding SET domain protein 1; KOA: Knee osteoarthritis; Sox9: SRY-box transcription factor 9; NC: Negative control; H&E: Hematoxylin and eosin; OARSI: Osteoarthritis Research Society International; ROS: Reactive oxygen species; DCFH-DA: 2′,7′-dichlorodihydrofluorescein diacetate; MDA: Malondialdehyde; GSH: Glutathione; qRT-PCR: Quantitative real-time polymerase chain reaction; Gpx4: Glutathione peroxidase 4; Acsl4: Acyl-CoA synthetase long-chain family member 4; Slc7a11: Solute carrier family 7 member 11; Mmp13: Matrix metalloprotease 13; ANOVA: Analysis of variance.

To evaluate the *in vivo* relevance of the *Nsd1*-*Sox9* axis in KOA, we suppressed *Sox9* expression in mice using siRNA constructs (*P* < 0.01, Figure 6A and 6B) and combined this with *Nsd1* overexpression. *Sox9* suppression exacerbated the pathological changes in the cartilage tissue of KOA mice, as evidenced by increased cartilage damage (Figure 6C), significantly higher OARSI scores (*P* < 0.01, Figure 6D), and elevated levels of ferroptosis markers in the cartilage (*P* < 0.05, Figure 6B and 6E–6H). These findings indicate that inhibition of *Sox9* partially reverses the protective effects of *Nsd1* overexpression on KOA symptoms in mice.

## Discussion

**Figure 7.**
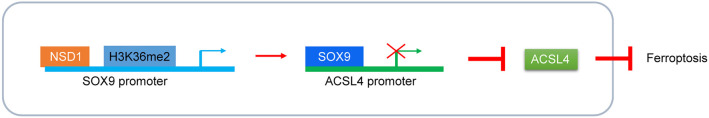
**NSD1 promotes *SOX9* expression by enhancing H3K36me2 modification on the *SOX9* promoter, thereby facilitating the transcriptional repression of *ACSL4* by SOX9 and subsequently suppressing *ACSL4* expression, leading to the inhibition of cellular ferroptosis.** NSD1: Nuclear receptor-binding SET domain protein 1; H3K36me2: Dimethylate lysine 36 of histone 3; SOX9: SRY-box transcription factor 9; ACSL4: Acyl-CoA synthetase long-chain family member 4.

The management of KOA remains challenging, particularly due to the potential side effects associated with current treatment methods [22]. However, recent studies suggest that targeting ferroptosis-related molecules and signaling pathways in chondrocytes may offer a promising therapeutic strategy for KOA [23, 24]. In our study, we uncovered a novel mechanism whereby NSD1 promotes *SOX9* expression by enhancing H3K36me2 modification on the *SOX9* promoter. This upregulation of *SOX9* subsequently inhibits *ACSL4* expression and ferroptosis in chondrocytes, pointing to a potential new treatment avenue for KOA (Figure 7).

Following the induction of KOA in mice, we observed significant structural damage and fibrosis in cartilage tissues, characterized by irregularities in the AC and a marked decrease in *Nsd1* expression. Previous studies have shown that *Nsd1* expression is downregulated in cartilage cells subjected to tensile stress, leading to decreased levels of H3K36 methylation and accelerating the progression of OA [25]. Additionally, *Nsd1* has been implicated in mediating the transfer of *Osr2* within the joint, thereby contributing to the maintenance of AC homeostasis [10]. In our study, overexpression of *Nsd1* in KOA mice resulted in significant improvements in cartilage structure, reduced fibrosis, and lower OARSI scores, alongside the inhibition of ferroptosis.

While there have been no prior reports specifically linking *Nsd1* to the regulation of ferroptosis, our findings are the first to demonstrate that *Nsd1* overexpression in cartilage tissues can alleviate ferroptosis, offering a novel therapeutic direction for KOA treatment. This effect was further corroborated by our *in vitro* experiments, where *NSD1* overexpression in IL-1β-treated chondrocytes restored cell viability, reduced levels of intracellular ROS, Fe^2+^, MDA, and ACSL4, and increased levels of GSH and GPX4. Together, these results indicate that *NSD1* overexpression can mitigate KOA by inhibiting chondrocyte ferroptosis both *in vivo* and *in vitro*.

Our ChIP results demonstrated the enrichment of NSD1 and H3K36me2 on the *SOX9* promoter, confirming that this increased enrichment correlates with elevated transcription and protein levels of *SOX9*. Epigenetic regulation of *Sox9* through histone modifications, such as methylation and demethylation, is known to play a crucial role in chondrocyte differentiation [26, 27]. Increased *Sox9* protein levels have been shown to activate chondrocyte anabolism in injured cartilage [28], and overexpression of *SOX9* has been reported to mitigate the inhibitory effects of TNF-α on chondrocyte growth and apoptosis induced by IL-1β [29]. Notably, reduced *Sox9* expression in chondrocytes leads to increased ECM degradation, ROS accumulation, and heightened sensitivity to ferroptosis [30]. For instance, treatment with icariin has been shown to restore type II collagen and *SOX9* expression in chondrocytes undergoing ferroptosis, while also reducing ROS and MDA levels and increasing GPX4 expression [14]. Our experiments were consistent with these findings, as we observed that inhibition of *SOX9* resulted in decreased chondrocyte activity and reversed the molecular changes related to ferroptosis that were regulated by *NSD1* overexpression. Furthermore, inhibiting *Sox9* *in vivo* exacerbated pathological changes in mouse cartilage tissue, significantly increased OARSI scores, and elevated levels of ferroptosis. Although the role of *Sox9* in chondrocyte ferroptosis in OA has been previously reported [31], specific mechanistic insights have been lacking. Our study offers a novel perspective by proposing that NSD1 regulates *SOX9* through H3K36me2, thereby influencing chondrocyte ferroptosis. In summary, the overexpression of NSD1 enhances H3K36me2 on the *SOX9* promoter, which alleviates KOA symptoms and chondrocyte ferroptosis, while *SOX9* inhibition reverses these effects.

Additionally, analysis using the JASPAR database identified a binding site for SOX9 on the *ACSL4* promoter. Substantial evidence supports that overexpression of *ACSL4* promotes ferroptosis in chondrocytes [23, 32]. In ferroptotic chondrocytes, elevated *ACSL4* protein levels are associated with reduced mRNA levels of Col2a1, Acan, and GPX4 [32]. GPX4, an essential antioxidant enzyme, plays a protective role in ferroptosis by directly scavenging lipid peroxides and indirectly regulating iron metabolism and redox status [33]. Acan and Col2a1 are critical components of the cartilage ECM [34], and ECM degradation is a hallmark of OA cartilage tissue [35]. These findings suggest that *ACSL4* not only promotes ferroptosis in OA cartilage cells but may also be linked to ECM degradation in OA cartilage. However, further research is needed to validate this connection. Our current study found that SOX9 binds to the *ACSL4* promoter and inhibits its transcription, thereby suppressing ferroptosis in chondrocytes.

Our study has several limitations that warrant consideration. The histone modifications mediated by NSD1 may not be exclusive to *SOX9*, as they could potentially regulate the expression of other genes involved in ferroptosis within chondrocytes. We focused exclusively on ferroptosis in chondrocytes, without investigating its role in other cell types, such as osteoblasts and osteoclasts, which may also play crucial roles in the progression of KOA. Additionally, due to budget constraints, we were unable to perform staining of synovial tissue, which could have provided further insights into the broader impact of NSD1 on KOA. It also remains unclear whether NSD1 is involved in other forms of cell death, such as autophagy or necroptosis, during KOA progression. Future studies should aim to identify other downstream target genes of NSD1 and explore their involvement in various cellular processes and other cell types during KOA development.

## Conclusion

In conclusion, our study demonstrates that NSD1 enhances *SOX9* expression through increased H3K36me2 modification, leading to the suppression of *ACSL4* expression and inhibition of ferroptosis in chondrocytes. These findings suggest that NSD1 may serve as a potential therapeutic target for the treatment of KOA in the future.

## Data Availability

The datasets generated during and/or analyzed during the current study are available from the corresponding author upon reasonable request.
